# Endotypes of Paediatric Cough—Do They Exist and Finding New Techniques to Improve Clinical Outcomes

**DOI:** 10.3390/jcm13030756

**Published:** 2024-01-28

**Authors:** Hannah E. O’Farrell, Hing Cheong Kok, Suhani Goel, Anne B. Chang, Stephanie T. Yerkovich

**Affiliations:** 1 NHMRC Centre for Research Excellence in Paediatric Bronchiectasis (AusBREATHE), Child and Maternal Health Division, Menzies School of Health Research, Charles Darwin University, Darwin, NT 0810, Australia; hingcheong.kok@menzies.edu.au (H.C.K.); anne.chang@menzies.edu.au (A.B.C.); stephanie.yerkovich@menzies.edu.au (S.T.Y.); 2 Australian Centre for Health Services Innovation, Queensland University of Technology, Brisbane, QLD 4000, Australia; suhani_goel@hotmail.com; 3 Department of Paediatrics, Sabah Women and Children’s Hospital, Kota Kinabalu 88996, Sabah, Malaysia; 4 Department of Respiratory and Sleep Medicine, Queensland Children’s Hospital, Brisbane, QLD 4101, Australia

**Keywords:** cough, children, review, endotypes, multi-omics, biomarkers

## Abstract

Chronic cough is a common symptom of many childhood lung conditions. Given the phenotypic heterogeneity of chronic cough, better characterization through endotyping is required to provide diagnostic certainty, precision therapies and to identify pathobiological mechanisms. This review summarizes recent endotype discoveries in airway diseases, particularly in relation to children, and describes the multi-omic approaches that are required to define endotypes. Potential biospecimens that may contribute to endotype and biomarker discoveries are also discussed. Identifying endotypes of chronic cough can likely provide personalized medicine and contribute to improved clinical outcomes for children.

## 1. Introduction

Chronic cough is the most common symptom of many conditions, such as bronchiectasis, which is a serious and neglected disease [1,2]. Both bronchiectasis [1] and chronic cough have large unmet needs, in addition to their underappreciated burden and importance [3,4,5]. As chronic cough is a heterogeneous clinical phenotype associated with several conditions, there is a need for better disease characterization to provide diagnostic certainty and to allow the development of novel and targeted therapies. This review aims to explore the current data on endotypes of chronic cough and the potential use of multi-omics in disease characterization that can improve the clinical outcomes for children with chronic cough.

## 2. What Are Endotypes

Disease characterization includes identifying phenotypes (observable characteristics linked to clinical outcomes, e.g., those with frequent exacerbations) and endotypes (patient groups with distinct underlying pathobiological mechanisms linked to a clinical phenotype or treatment responses). Broadly, endotypes can be based on microbial data or non-microbial types. For example, in the field of chronic cough in children, cross-sectional data have described that airway microbiota could discriminate diagnostic groups (controls, children with protracted bacterial bronchitis [PBB] or bronchiectasis) [6]. Whilst data on other microbial-based endotypes of airway disease are scarce in children, there are much more such data in adults [7]. There are vastly different types of non-microbial-based endotypes, and these include those based on type of inflammation, anatomical abnormality and gene expression. The latter have already positively impacted clinical care in diseases like breast cancer [8]. Therefore, identifying and validating relevant endotypes of paediatric cough is essential in improving clinical outcomes and individualized disease management. This article focuses on non-microbial-based endotypes, as there is another review article on the former.

## 3. Endotypes in Different Airway Diseases

Currently there are no endotypes that can differentiate between the many causes of chronic cough in children. Hence, we focus this review on endotypic descriptions of the common causes of chronic cough in children and present what is available in adults [9].

### 3.1. Asthma Endotypes

Asthma is a chronic inflammatory airway disease characterized by symptoms of variable airflow obstruction (recurrent wheeze and dyspnea and sometimes cough) and bronchial hyper-responsiveness, demonstrated through a pulmonary function test. Asthma is a heterogeneous disease with different phenotypes and endotypes. One of the most commonly described endo-phenotypic classifications is based on the underlying airway immune-mediated inflammation, i.e., type 2 (T2)-high eosinophilic-driven asthma and T2-low classifications with various biomarkers used, including sputum cellular inflammation. Currently, the field of endotypic classification of respiratory disorders is the most advanced in asthma compared to other chronic airway diseases. Several published reviews have covered asthma endotypes in detail [10,11,12]. 

There are now a myriad of therapeutic options with effective anti-inflammatory drugs, short- and long-acting beta2 agonists, short- and long-acting muscarinic receptor antagonist and biologics. The management of asthma has changed fundamentally in those with severe asthma with the availability of biologics. These targeted treatments are based on measurements of total serum immunoglobulin (Ig)-E, blood eosinophil counts and fractional concentration of exhaled nitric oxide (FeNO) that enable the identification of specific T2-high endotypes responsive to specific biologic therapies [13]. Four distinct sputum cellular inflammatory phenotypes have been identified in adults with asthma: eosinophilic asthma, neutrophilic asthma, mixed granulocytic asthma and paucigranulocytic asthma [14]. The classification can be further simplified to eosinophilic and non-eosinophilic, with the latter group consisting of neutrophilic and paucigranulocytic cellular patterns in the sputum. 

In adults with asthma, systemic (blood) gene expression signatures and/or pro-inflammatory proteins are detected in the presence of inflamed airways [15,16]. Airway gene expression signatures can discriminate inflammatory phenotypes, predict corticosteroid responsiveness [17] and identify exacerbations in prone adults better than traditional methods [15,16]. A sputum 6-gene signature can be used to predict future exacerbations of poorly controlled asthma in adults [18]. To date, no such data are available for children. 

Sputum cellularity is one of the most widely studied airway inflammatory markers that was developed over two decades ago [19,20]. The development of a sputum gene expression signature can further discriminate these inflammatory phenotypes seen in asthma [21]. The sputum pheno-endotype is relatively stable in adults, although some studies have suggested variability [22]. In contrast, the data differ in children with asthma, where sputum cellularity is unstable, and therefore, the endotype will also be unstable. Conversely, in children with asthma, sputum inflammatory phenotypes are variable in both the stable and exacerbation phases, which contrasts with adult data [23]. In a small prospective study, 13 of 32 children (41%) with stable asthma demonstrated a change in the sputum inflammatory phenotype eight weeks later [23]. Another study showed that the percentage of both sputum eosinophils and neutrophils increased and peaked on day one of an asthma exacerbation compared to baseline, but 22% (2/9) and 13% (1/8) of children had sputum phenotype categorization changes on day one and day three of exacerbation, respectively [23].

While progress has been made in defining asthma endotypes, one of the key issues is the variability in the clinical phenotypes and the need to expand beyond the known T2 biomarkers, as T2 inflammation cannot be accurately predicted on the basis of serum markers alone [24]. To provide a more detailed clinical picture, future endotyping of disease phenotypes in children will also need to explore different biologic samples, as not all children can produce sputum.

### 3.2. Bronchiectasis Endotypes

Bronchiectasis is a clinical syndrome characterized by recurrent or persistent wet/productive cough, with airway inflammation and infection as key contributors to its disease pathophysiology [25]. It is confirmed by an abnormal bronchial dilatation on chest computer tomography scans [25]. Adult bronchiectasis data on phenotypes (e.g., frequent exacerbators) and endotypes (airway microbiology and inflammation) have begun to have a clinical impact [26]. Adult bronchiectasis phenotype data can inform prognosis, e.g., frequent exacerbators have poorer outcomes (hospitalisation and death) [27]. There are also emerging adult bronchiectasis endotypes based on inflammatory profiles associated with future exacerbation risk [28]. Neutrophilia is the predominant inflammatory endotype [29], and those with this dominance may benefit from brensocatib, an inhibitor of neutrophil serine proteases [26]. Airway or peripheral eosinophilia [30] has more recently been described and is an endotype where corticosteroids will likely be beneficial [31]. 

For childhood bronchiectasis, we have proposed treatable traits [32], but currently no published prospective data exist. A retrospective study based on the Australian Bronchiectasis Registry found the frequent exacerbator phenotype to be younger (ORadj 0.9, 95% CI 0.8–0.9), more recently diagnosed (ORadj 0.7, 95% CI 0.6–0.8), and to harbor *P. aeruginosa* (ORadj 2.4, 95% CI 1.0–5.8) [33]. No specific non-bacterial endotypes were examined in that study. Paediatric data are required, as it is known that substantial clinical differences between children and adults exist in this field. Specifically, with respect to endotypes, it is known that bacterial microbiota [34] and pathogen [32] data from older adults with bronchiectasis are significantly different to those of children, while immunological responses (as reflected in gene expression) are age dependent.

### 3.3. PBB Endotypes

PBB was first described by our group [35] and is now an internationally recognized diagnostic entity [36,37]. Chronic wet cough is a key symptom of PBB and bronchiectasis. Both are associated with lower airway bacterial infection and inflammation [35], and it is now recognized that PBB starts a continuum of clinical and pathobiological features that ends with bronchiectasis. Children with recurrent episodes of PBB and *Haemophilus influenzae* infections confirmed by bronchoalveolar lavage (BAL) are more likely to develop bronchiectasis [38]. While we have shown that airway cells in PBB and bronchiectasis share similar inflammatory [39] and sphingosine-1-phosphate [40] gene expression profiles, there are no endotypes that distinguish between PBB and bronchiectasis. In the future, it will be important to identify PBB endotypes that could distinguish children at risk of bronchiectasis and poor outcomes and those more likely to respond to treatment.

### 3.4. Pneumonia Endotypes

Pneumonia is typically diagnosed with clinical symptoms/signs and confirmed by chest radiography. Many host biomarkers have been thoroughly investigated for their association with pneumonia diagnosis, disease severity and mortality. Adult studies [41] showed that lipopolysaccharide-binding protein, interleukin-6 (IL-6) and IL-2 receptor were specific features for community-acquired pneumonia (CAP). Another adult study [42] highlighted that lymphopenia alone rendered a 3.8-fold risk of mortality in patients with severe CAP with septic shock. However, the opposite is true in the paediatric population. Florin et al. showed that white blood cell count, C-reactive protein or procalcitonin used in isolation are not helpful in discriminating overall disease severity in children with CAP [43].

Currently, data are scarce for host-focused endotyping for pneumonia. One possible endotype of pneumonia suggested is immunoparalysis, which is based upon the interpretation of blood transcriptomic levels [44]. Davenport et al. [45] found two sepsis response signatures (SRS1 and SRS2) from the transcriptomic analysis of peripheral blood leucocytes of adult patients with sepsis due to CAP. SRS1 was characterized by endotoxin tolerance, T-cell exhaustion and human leucocyte antigen class II downregulation and was associated with a higher 14-day mortality than SRS2. Subsequently, Scicluna et al. [46] discovered four molecular endotypes of blood leucocyte genome-wide expression profiles from a cohort of adult and paediatric patients with sepsis caused by CAP and called the molecular diagnosis and risk stratification of sepsis 1−4 (Mars1−4). Of these, three endotypes, Mars1, Mars2 and Mars4, were detected with favorable stability in children. The Mars1 endotype was also associated with the highest mortality in septic patients compared with the Mars2−4 endotypes. Nevertheless, more discoveries are needed for the specific endotyping of children with pneumonia so that host-directed clinical approaches can be achieved for predicting, preventing and treating pneumonia.

### 3.5. Tuberculosis Endotypes

Tuberculosis (TB) is a chronic infection resulting in immune suppression [47]. Adults and children have a distinction in susceptibility to Mycobacterium tuberculosis (Mtb) infection due to age-related differences in their immunological response [48]. TB in children has a broad spectrum of clinical presentations, from non-specific symptoms to pulmonary TB and extra-pulmonary manifestations (e.g., TB meningitis). These variable phenotypes are caused by several specific endotypes that lead to TB disease, with three mutually non-exclusive TB endotypes proposed [49]. The first involved defects in the interleukin-12–interferon-gamma (IL-12–IFN-γ) signaling pathway. The upstream mutations in the IFN-γ pathway caused a reduction in IFN-γ, and the downstream defects in the IFN-γ receptors caused signal transduction failure. Both processes impaired the capacity of host cells to kill intracellular Mtb. Second, an exuberant hyperinflammation of tumor necrosis factor (TNF) and IFN-γ led to macrophage necrosis and the escape of viable Mtb into the extracellular space. The third proposed endotype involved immune exhaustion due to chronic antigenic stimulation, resulting in the decreased production of cytokines (TNF, IL-2 and IFN-γ).

A more recent classification of TB endotypes based on unbiased bioinformatic techniques has been published [50]. They identified two main TB endotypes: A and B. Endotype A had an increased expression of genes related to inflammation and immunity and a decreased expression of genes related to metabolism and proliferation. Endotype B had an increased activity of genes that regulate metabolism and proliferation pathways. In addition, TB endotype A showed characteristics of immune exhaustion, i.e., hyperinflammatory coupled with hyporesponsiveness. The study also found that TB endotype A had reduced clinical cure rates and increased mortality compared to endotype B. While these findings were mainly from adult data, the lack of effective host-focused endotyping for paediatric TB is a major shortcoming. A South African, case-control, birth cohort study (The Drakenstein Child Health Study) [51] indirectly addressed this knowledge gap by examining the relationship between gene expression profiles from umbilical cord blood and tuberculin skin test conversion and TB disease in the first five years of life. They found several novel gene expression profiles associated with tuberculin conversion and progression to TB disease among children with early infection, providing novel endotype data in children.

### 3.6. COPD Endotypes

Chronic obstructive pulmonary disease (COPD) is predominantly an adult lung condition punctuated by exacerbations of respiratory symptoms. Distinct endotypes of COPD have been identified that are related to different causal genetic mechanisms, including alpha-1 antitrypsin deficiency and telomerase polymorphisms, with inflammatory endotypes also recognized in stable and exacerbating COPD that have implications for the choice of therapy.

COPD patients have chronic inflammation in the lungs, characterized by increased sputum neutrophils, and not surprisingly, emerging inflammatory COPD endotypes have been described. Endotypes characterized by COPD exacerbations show patterns similar to stable-state inflammatory endotypes, with several studies reporting subgroups defined as (1) pro-inflammatory corresponding to bacterial infection; (2) T1 inflammation corresponding to viral infections; (3) T2 inflammation corresponding to eosinophilic inflammation; and (4) pauci-inflammation [52,53,54,55]. Particular attention has been paid to eosinophilic COPD with T2-mediated airway inflammation [56], as it is associated with a greater risk of exacerbation [57], and this exacerbation risk is reduced with inhaled corticosteroid treatment [58]. Blood eosinophil counts have shown longitudinal variability, and those with the highest variability have more frequent exacerbations compared to those with a stable blood eosinophil count [59].

Clinical trials targeting IL-5 (mepolizumab and benralizumab) and IL-4/IL-13 (dupilumab) [60,61,62,63,64] have shown mixed efficacy, but further sub-analyses of the trials indicated that only T2-driven COPD exacerbations were more likely to be suppressed with treatment. This highlights the need for continued endotyping of COPD exacerbations and the discovery of better biomarkers to provide more personalized treatment and guide therapeutic decisions.

### 3.7. Summary of Section

While it not possible to present a complete review of endotypes or unique phenotypes in all lung disease (e.g., that in bronchopulmonary dysplasia [65], interstitial lung disease [66] and psychogenic cough [67]), overall, endotyping of lung disease in children is in its infancy. There is a clear need for research that can produce robust data. While data obtained in adults can inform childhood diseases, it is necessary to obtain children-specific data, as there are clear differences between children and adults that impacts clinical management and outcomes. For example, the management of chronic cough in children with asthma differs from that in adults [68], and the concept of cough hypersensitive syndrome, which is commonly used in adults, is inappropriate in children [69].

Identifying ‘treatable traits’ through endotyping will enable a mechanistic approach to disease stratification, treatment and management, improving translation into clinical practice and advancing precision medicine [70]. Biomarkers are measurable indicators linking an endotype with a phenotype. One problem with endotype-driven research is that while endotypes can be linked to a single molecular mechanism, most share etiological and pathogenic pathways that may not be present in all patients of a specific disease subset or at a specific disease timepoint [70]. While the identification of novel biomarkers has improved the predicted response to treatment for certain diseases by tailoring disease management [71], no biomarkers currently exist that are precise enough to identify a specific endotype for paediatric cough. Furthermore, given the heterogeneity of conditions associated with chronic cough, it is likely that a combination of biomarkers will be required for discriminatory power.

## 4. Multi-Omics Approaches for Biomarker Discovery in Respiratory Disease

A multi-omics approach is now preferred in biomarker discovery research, as it allows for a more comprehensive and integrated analysis of biological systems and disease mechanisms [72,73]. There are three categories of multi-omics approaches: (1) ‘genome first’, which determines gene loci mechanisms contributing to disease; (2) ‘phenotype first’, which focuses on the pathways contributing to disease; and (3) ‘environment first’, which analyses how the environment interacts with genetic variation, affecting downstream disease pathways [71,72]. By analyzing multiple omics data types, including genomics, transcriptomics, proteomics and metabolomics, researchers can obtain a more complete understanding of how different molecules and pathways interact and how they could potentially contribute to disease processes, especially for complex diseases where the etiology is far more intricate with no clear deterministic factor [72]. Indeed, integrating omics outcomes with clinical data has enabled the identification of endotypes associated with a higher risk of asthma development after bronchiolitis [74,75]. Additionally, regardless of the approaches used to identify novel disease biomarkers, diagnostic accuracy needs to be validated following international recommendations, such as the Standards for Reporting Diagnostic Accuracy (STARD) guidelines [76]. 

Current challenges with omics approaches include disease insights being mostly comparative, where differences in data from healthy and diseased populations are assumed to be directly related to disease. However, complex phenotypes bring heterogeneity [72]. This heterogeneity can arise from sample ascertainment, batch effects, population structure, cell type composition bias and merging datasets with different analysis methods, among other unknown factors [71,72]. Therefore, multi-omics techniques will need to be performed using large datasets and from samples across multiple timepoints and institutions to identify endotypes of paediatric cough with clinically translatable biomarkers. All omics data will need to be generated from single samples using standardized protocols, which will help reduce heterogeneity complications overall and enable more uniform data comparisons and statistical analyses. 

### 4.1. Genomics 

The field of genomics is the study of the total or a part of the genetic or epigenetic sequence information of organisms, and it is the most mature of all multi-omics approaches [77]. This approach focuses on the identification of genetic variants associated with disease. As deoxyribonucleic acid (DNA) from non-cancerous somatic cells is relatively stable over time, it can be used to measure an individual’s variation across the genome and their lifetime, with genetic testing now integrated into clinical practice [77,78]. In the early 2000s, efforts to catalogue common DNA single nucleotide polymorphisms (SNPs) led to commercially available microarrays, with scientists relating common SNPs to disease through genome-wide association studies (GWAS) [78,79]. Genetic testing was further advanced with next-generation sequencing technologies (whole-exome and whole-genome sequencing), which enabled the characterization of an individual’s entire genome [78]. 

For more complex heterogeneous diseases, large sample sizes are required, which has prompted investigators to pool cohorts to obtain good statistical power and detect significant disease associations. Consortium projects have now been published for COPD, including ECLIPSE [80], COPDGene [81] and ICGN [82], as well as for asthma, including GABRIEL [83] and the EVE Asthma Genetics Consortium (https://eve.uchicago.edu/, accessed on 8 November 2023). 

### 4.2. Transcriptomics

The transcriptome comprises the entire set of ribonucleic acid (RNA) species or transcripts in a given cell, tissue or organism, including coding RNA (translated into proteins) and non-coding RNA (involved in post-transcriptional control and regulates gene expression). Unlike the genome that remains relatively stable over one’s lifetime, the transcriptome is variable. It can provide informative snapshots of the total transcripts or gene expression changes present in a cell and how these functional changes contribute to disease development [84].

The early 1990s saw the first attempts at studying the whole transcriptome, with rapid technological advances since, further highlighting transcriptomics as an established discipline. The two main transcriptomic techniques include microarrays, which quantify a predetermined set of RNA transcripts/genes through hybridization and RNA sequencing (RNA-seq), which uses high-throughput sequencing and computational methods to capture all RNA transcripts [84]. Due to reduced costs and increased sensitivity and specificity, RNA-seq has overtaken microarrays as the dominant transcriptomic technology [85].

Identifying novel biomarkers using transcriptomics requires the identification of major changes in gene or transcript expression under disease or controlled conditions. The RNA needs to be collected from an appropriate sample type and site, as cells and tissues have characteristic transcriptomes [86]. The most appropriate sites and sample types for transcriptomic studies in respiratory diseases include blood, sputum, BAL fluid and lung and airway tissue. Additional details on these samples will be elaborated upon in this review. 

Several transcriptomic studies have identified disease-specific pathways and relevant genes. Combined with clinical variables, these have further identified expression signatures characterizing groups of patients into sub-phenotypes and endotypes [78,87]. Using peripheral blood from the Unbiased Biomarkers in Prediction of Respiratory Disease Outcomes (U-BIOPRED) cohort study, researchers reported that peripheral blood gene signatures from asthma patients could determine disease severity and response to oral steroids [88]. A sputum transcriptomics analysis of severe asthma patients identified four stable groups with distinct clinical characteristics, of which the genes identified in these groups were potential biomarkers for group-specific treatment [89]. 

### 4.3. Proteomics

Proteomics is the study of the entire protein profile of an organism, including structure and function. This provides a more comprehensive insight into cells’ metabolic state and physical interactions compared to genomics, transcriptomics and epigenomics [90]. While proteomics provides in-depth cross-sectional time and space snapshots of protein levels, assays can also measure protein localization, post-translational modifications and protein-protein interactions [90]. Like microarray technologies to measure gene expression, protein microarrays were first used for protein biomarker discovery works, which have now advanced with high-throughput technologies, including mass spectrometry techniques and gel-based techniques. 

There are three major types of proteomics, which include (1) expression proteomics (measures protein expression between disease and control patients using quantitative and qualitative methods); (2) functional proteomics (determines 3D structure and complexities of functional proteins using nuclear magnetic resonance and X-ray crystallography); and (3) structural proteomics (investigates the biological role of proteins and their interactions within a cell) [90]. In addition, there are two main proteomic workflows, which include (1) top-down (mass spectrometry technique that analyses intact proteins) and (2) bottom-up (measures digested peptide mixtures through column separation, followed by mass spectrometry analysis) [90,91].

Proteomic studies using plasma, sputum and lung tissue have identified potential protein biomarkers distinguishing asthma patients and COPD patients from controls [92,93], as well as nonsmokers, smokers and mild COPD compared to those with severe COPD [94]. These studies highlight the strength of proteomics for biomarker discovery and that identifying novel endotypes for paediatric cough should be considered a priority omics technology. 

### 4.4. Metabolomics

Metabolomics is the study of the complete collection of metabolites (small molecules < 1 kDa) and how they participate in chemical reactions [78,95]. Nuclear magnetic resonance and high-resolution mass spectrometry are the most common techniques used to characterize the metabolome [78]. Analysis of the metabolome is considered one of the most comprehensive omics approaches, as it can highlight the metabolic reactions that have already occurred, reflecting the biological phenotype of a sample and whether this was caused by disease state, environment, diet or genetic mutations [96]. 

An advantage of metabolomics is that the sample types that can be assessed are minimally invasive, including urine, exhaled breath condensate (EBC) and blood [78]. This is particularly appealing for paediatric studies, where sample volumes are often small and difficult to obtain. Metabolic markers have been identified from these samples in children with asthma [97,98] and adult patients experiencing an exacerbation of COPD [99]. However, they remain to be validated in independent patient cohorts. Metabolomics holds great promise in identifying novel endotypes for paediatric cough, particularly from non-invasive sample types. 

## 5. Biospecimens for Biomarker Measurement for Respiratory Diseases in Children 

The measurement of a clinically significant biomarker, especially from paediatric patients, needs consideration that the sample type is readily available, measurable, non-invasive, cost effective and corresponds with age-dependent physiologic changes [100]. Biospecimens used to assess respiratory disease include lung tissue, BAL fluid, sputum and blood, with non-invasive samples including exhaled breath, urine and saliva. Access to sample types such as lung tissue and BAL are limited in children due to their invasive nature, and large volumes of blood and sputum (with young children generally unable to expectorate) often cannot be obtained. Therefore, there is an urgent need to discover methods to obtain other biofluids that are more readily available and easily obtainable in a non-invasive manner from children. 

### 5.1. Blood

Peripheral blood is a relatively non-invasive and easily stabilized specimen type for identifying gene expression signatures. Systemic (blood) gene expression signatures and/or pro-inflammatory proteins are detected in the presence of inflamed airways (e.g., TB and asthma [72,76]). Studies investigating blood gene expression signatures for COPD with clinical features have identified novel underlying disease mechanisms [101,102]. We have previously found that airway cells in PBB and bronchiectasis share similar gene expression profiles [39]. Additional omics technologies, including transcriptomics (RNA-seq), may allow us to further differentiate these patient groups and identify novel cough endotypes. 

Another advantage of blood for downstream omics analyses includes the usability of current collection and storage tubes. Technologies such as PAXgene™ (Becton Dickinson Pty Ltd., Franklin Lakes, NJ, USA) blood RNA tubes (PreAnalytiX, Hombrechtikon, Switzerland) contain an additive that stabilizes in vivo gene expression profiles [103]. As only a small volume of whole blood (2.5 mL) is required for collection, they are ideal for use in multicenter studies (for consistency) and for paediatric cohorts. PAXgene tubes also allow blood to be stored at room temperature for longer periods (up to 72 h), which should be considered for studies in rural and remote regions. 

### 5.2. BAL Fluid

BAL fluid has been considered the gold standard for clinicians to assess infection and inflammation in the lungs, with secretions providing an optimal source of inflammatory cells directly from the lower airway [104]. Research has shown that neutrophil elastase from BAL predicted pulmonary exacerbations in young children with cystic fibrosis [105]. BAL fluid has also been used to assess the lung transcriptome and proteome for a number of respiratory diseases, such as lung cancer, asthma, COPD and coronavirus disease 2019 (COVID-19) [106,107,108,109]. It provides insights into novel disease mechanisms and pathogenic pathways. However, to obtain BAL fluid from children, bronchoscopy needs to be performed under a general anesthetic by skilled physicians and is not readily available at many centers. Additionally, BAL specimens used for biomarker discovery are heavily diluted, making it difficult for the nucleic acid yield to pass the necessary quality control metrics for use in downstream omics technologies. Identifying gene signatures from BAL fluid samples using omics technologies may provide preliminary gene signatures that could then be validated in non-invasive sample types, such as saliva and urine.

### 5.3. Sputum

Unlike BAL fluid, expectorated sputum has been used as a less invasive alternative to measure airway inflammation [110]. The measurement of sputum neutrophil elastase has been suggested as a promising biomarker for bronchiectasis severity, with increased levels indicating the need for aggressive management [104]. However, most children have difficulties in expectorating, and most downstream assessments of sputum require a large yield. One technique to produce sputum is induction with nebulized hypertonic saline [104]. 

Researchers have been able to identify a sputum biomarker signature that can discriminate adult asthma inflammatory phenotypes [21], with further transcriptomics analyses identifying cellular pathways underlying inflammation and corticosteroid resistance, as well as molecular mechanisms [111]. Additionally, sputum gene expression signatures have been identified to predict COPD exacerbations [112]. While sputum is a relatively non-invasive sample type that has been a successful biomarker for identifying some respiratory diseases, it is not ideal for paediatric studies.

### 5.4. Breath 

Exhaled breath has gained much attention as a non-invasive respiratory disease biomarker, particularly for paediatric patients. FeNO is a marker of airway inflammation and is currently used for Th2-mediated asthma differentiation. It is also capable of predicting inhaled corticosteroid responsiveness [113]. Although FeNO is not a diagnostic marker of bronchiectasis, it can rule out the diagnosis in asthmatic patients because the levels are generally low in patients with bronchiectasis [114].This is possibly due to the prevalence of neutrophilic inflammation [115] in the lower airway.

EBC can be used to measure volatile organic compounds (VOCs) that originate from the airway lining fluid [116]. Studies have shown that inflammatory lung diseases such as cystic fibrosis, asthma and COPD can be diagnosed using exhaled VOCs [117,118,119]. There are two techniques that can be used to study exhaled breath VOC profiles: (1) a quantitative method of measuring individual components through gas chromatography with mass spectrometry and (2) a qualitative method using pattern recognition technology, such as electronic noses [120,121]. A multi-omics approach using EBC to measure VOCs may reveal disease-specific profiles and help distinguish paediatric cough endotypes. 

### 5.5. Extracellular Vesicles (EVs)

EVs are potent packages of concentrated cellular information (containing individual molecules, such as DNA, RNA, microRNAs [miRNAs], lipids and proteins). They have been shown to play a vital role in cell–cell communication, highlighted by their ability to exchange information with recipient cells [122]. Due to the ability of EVs to influence physiological and pathological conditions using their concentrated bioactive cargo, they are promising biomarkers that can be used to reflect disease states [122,123] compared to other biomarkers, such as circulating RNA, which is unstable and easily degraded by circulating ribonucleases (RNases) [122]. The composition of EVs in the lipid bilayer serves as a protective shield against degradation (circulating RNases’ ability to withstand multiple freeze–thaw cycles) and assists in dissemination throughout the body in a variety of bodily fluids (such as saliva and urine).

#### 5.5.1. Saliva EVs

Saliva’s use as a diagnostic tool has huge advantages, particularly for paediatric patients, mainly due to the collection being fast, inexpensive, non-invasive and risk-free [124,125]. Its use in microfluidic devices for point of care (POC) diagnostics is also considered ideal in field testing compared to blood and urine specimens. The latest microfluidics devices for POC saliva testing include ‘Lab-on-a-chip’ (LOC) devices, which use picolitre volumes of fluid to produce fluidic circuits through microfabrication to perform laboratory functions [126]. The recent COVID-19 pandemic highlighted the urgent clinical need for rapid POC diagnostic devices, and numerous LOC modalities for virus testing were developed [126]. 

Saliva is considered a surrogate to blood as a marker of systemic disease. The components found in blood are also found in saliva and can reflect physiological and pathological states of the body [125,127]. Various molecules, including enzymes, hormones, antibodies, antimicrobial components and nucleic acids, can transfer from blood to saliva through transcellular or paracellular transport [125]. It has been proposed that EVs may play a role in transporting RNAs of systemic origin from blood into saliva and may hold the key to improving saliva’s diagnostic capacity by concentrating nucleic information that is otherwise low in whole saliva [128]. 

#### 5.5.2. Urine EVs

Urine is an appealing biological sample for biomarker discovery, particularly for children, due to its ease and safety of collection. It is also non-invasive, and unlike other non-invasive biospecimens such as saliva, a large volume is readily available. Additionally, as renal filtration is part of urine formation, it is a less complex matrix compared to blood, with fewer factors known to interfere with biomarker assays [129]. Metabolites found in urine, including bromotyrosine and leukotriene E, have been identified as potential diagnostic biomarkers for asthma [130,131], as well as predictors of exacerbations and response to steroid treatments [132]. 

Urinary EVs have gained attention as a more concentrated source of potential biomarkers, as they are robust and stable. They contain disease-specific biomolecules, including DNA, RNA and proteins, reflecting their parent cell’s physiological state and microenvironment [122]. Urine profiling has identified biomarkers for lung cancer diagnosis and tumor specificity [133]. More importantly, urine EVs have also been shown to be a useful indicator for assessing allergic airway diseases in children, specifically measuring the bacteria-derived molecular cargo within urine EVs [134]. This highlights the potential for urine EVs to be used as a biospecimen to identify paediatric cough-specific endotypes.

### 5.6. Summary of Section

As highlighted throughout this review, identifying novel biomarkers will provide measurable indicators to link an endotype with a phenotype (chronic cough). Taking into consideration the heterogeneity of chronic cough, a combination of biomarkers will most likely be required. In the context of paediatric patients, it is imperative that future research focuses on minimally invasive and readily available biospecimens. Additionally, a combination of multi-omics technologies would allow for the simultaneous assessment of a single sample type and would provide a comprehensive and integrated picture that is required for the identification of relevant endotypes of paediatric cough. Once endotypes have been identified, they can then be assessed for response to standard and emerging treatments. Targeting these ‘treatable traits’ will allow for their future implementation into clinical practice.

## 6. Conclusions

Chronic cough is the most common symptom of many childhood lung conditions. While endotypes have yet to be described, they hold promise in contributing to diagnosis, disease stratification and personalized therapies by identifying novel biomarker(s) and informing our understanding of pathobiological pathways. The availability of such data at the point of presentation will allow for improved individualized assessment that can lead to better treatment and outcomes, e.g., discovering those at risk of poorer outcomes, leading to changes in clinical practices, including earlier intensive treatment. Precision medicine approaches will require state-of-the-art multi-omics technologies, and while this has yet to be realized for chronic cough, there is now progress. Furthermore, as the field expands, the use of minimally invasive samples with minimal volumes will be very attractive for paediatric respiratory conditions. Future progress will require an integration of basic mechanistic studies and clinical science, which together will lead to improved clinical outcomes for children with chronic cough.

## Data Availability

Not applicable.
